# Genetic differentiation and restricted gene flow in rice landraces from Yunnan, China: effects of isolation-by-distance and isolation-by-environment

**DOI:** 10.1186/s12284-021-00497-6

**Published:** 2021-06-15

**Authors:** Di Cui, Cuifeng Tang, Hongfeng Lu, Jinmei Li, Xiaoding Ma, Xinxiang A, Bing Han, Yayun Yang, Chao Dong, Feifei Zhang, Luyuan Dai, Longzhi Han

**Affiliations:** 1 Institute of Crop Sciences, Chinese Academy of Agricultural Sciences, No. 12 Zhongguancun Nandajie, 100081 Beijing, China; 2 Institute of Biotech and Germplasm Resources, Yunnan Academy of Agricultural Sciences, No. 9 Xueyunlu, 650205 Kunming, Yunnan, China; 3 Novogene Bioinformatics Institute, 100083 Beijing, China

**Keywords:** Rice landraces, Genetic diversity, Genetic differentiation, Isolation-by-distance, Isolation-by-environment

## Abstract

**Background:**

Understanding and identifying the factors responsible for genetic differentiation is of fundamental importance for efficient utilization and conservation of traditional rice landraces. In this study, we examined the spatial genetic differentiation of 594 individuals sampled from 28 locations in Yunnan Province, China, covering a wide geographic distribution and diverse growing conditions. All 594 accessions were studied using ten unlinked target genes and 48 microsatellite loci, and the representative 108 accessions from the whole collection were sampled for resequencing.

**Results:**

The genetic diversity of rice landraces was quite different geographically and exhibited a geographical decline from south to north in Yunnan, China. Population structure revealed that the rice landraces could be clearly differentiated into *japonica* and *indica* groups, respectively. In each group, the rice accessions could be further differentiated corresponded to their geographic locations, including three subgroups from northern, southern and middle locations. We found more obvious internal geographic structure in the *japonica* group than in the *indica* group. In the *japonica* group, we found that genetic and phenotypic differentiation were strongly related to geographical distance, suggesting a pattern of isolation by distance (IBD); this relationship remained highly significant when we controlled for environmental effects, where the likelihood of gene flow is inversely proportional to the distance between locations. Moreover, the gene flow also followed patterns of isolation by environment (IBE) whereby gene flow rates are higher in similar environments. We detected 314 and 216 regions had been differentially selected between Jap-N and Jap-S, Ind-N and Ind-S, respectively, and thus referred to as selection signatures for different geographic subgroups. We also observed a number of significant and interesting associations between loci and environmental factors, which implies adaptation to local environment.

**Conclusions:**

Our findings highlight the influence of geographical isolation and environmental heterogeneity on the pattern of the gene flow, and demonstrate that both geographical isolation and environment drives adaptive divergence play dominant roles in the genetic differentiation of the rice landraces in Yunnan, China as a result of limited dispersal.

**Supplementary Information:**

The online version contains supplementary material available at 10.1186/s12284-021-00497-6.

## Background

Rice, as one of the world’s major food crops, feeds more people than any other crop species and is a staple crop for nearly all of Asia. Historically, rice yield has accounted for 40 % of the total grain output in China (Hu et al. 2002). Over the past 30 years, the phenomenon of large areas planted with single breeding varieties has increased the use of improved rice varieties, which has led to a narrow genetic base and an overall reduction in the genetic diversity of rice varieties grown in China (Xu et al. 2016).

Compared with improved varieties, traditional landraces are the products of natural and artificial selection that occurred over thousands of years. Landraces are grown by local farmers, and have been passed down from generation to generation. Landraces are defined as ‘‘geographically or ecologically distinctive populations (Pusadee et al. 2009), which are conspicuously diverse in their genetic composition both between landraces and within them,’’ (Brown 1978) and they are each identifiable by their unique morphologies and well-established local names (Harlan 1992). Landrace diversity is found throughout the cultivated range for rice, making the landraces a rich source of genetic variation that includes such characteristics as high grain quality, wide adaptability, strong environmental tolerance, disease and insect resistance, and cultural uses, and can provide a valuable gene pool for the discovery and utilization of favorable genes. However, over the past 50 years, local rice landraces have been largely replaced by genetically uniform, high-yielding modern varieties in many parts of China. Rice landraces are generally no longer planted in China, although there are exceptions in some ethnic minority regions in Yunnan or Guizhou.

Yunnan province is one of the important centers of genetic diversity for cultivated rice in China and throughout the world (Zhang et al. 2006). Over thousands of years of planting history, natural and artificial selection has preserved a large number of traditional rice landraces in Yunnan, due to its complex geographical environment, diverse climatic conditions, and rich national culture. A remarkably diverse set of rice landraces (including all varieties of *Oryza sativa* L. in China) are found in Yunnan, and include both *indica* and *japonica* types, those with glumes, with or without hairs, non-glutinous or glutinous, upland or lowland, naked rice, and rice with various hull grain colors (white, red, and purple) and flavors (ordinary or fragrant) (Zeng et al. 2000a; Zeng et al. 2000b).

By the late 1980s, there were 5,128 local rice landraces collected and catalogued in Yunnan, China. At present, many traditional rice landraces are still planted by indigenous farmers in the ethnic minority regions of Yunnan. These landraces have been passed down from generation to generation despite the availability of modern improved varieties, mainly due to reasons associated with the diversity of the local agro-ecology and also to fulfill cultural requirements (Xu et al. 2014). Indigenous farmers work to conserve the diverse traditional rice landraces on the farm, preferring not only highly productive landraces, but also those that are more resistant to diseases and pests and that display tolerance to extreme environmental conditions, as well as landraces that are of cultural importance (e.g., ethnic dietary customs, medicinal uses, festivals, and religious ceremonies) (Gao 2003). The rice landraces that are grown have desirable features, and they cannot easily be replaced by modern improved varieties because some of these landraces have been cultivated for > 50 years.

Rice landraces in Yunnan province are widely distributed over a region extending from 21° 8’ 32’’ N to 29° 11’ 18’’ N and 97° 31’ 39’’ E to 106° 11’ 47’ E (Zeng et al. 2007), and are planted at altitudes ranging from approximately 76.4 m in Hekou county to 2,695 m in Ninglang county in Yunnan (Xu et al. 2016). The heterogeneity of the environments and wide distribution of rice landraces provides an excellent opportunity for studies of genetic differentiation among populations in different geographical locations as well as for determining how geographical distance and environmental factors affect population genetic differentiation. Many studies have focused on population structure and genetic differentiation of rice landraces (Xiong et al. 2010; Xiong et al. 2011; Zeng et al. 2001). However, little is known about how geographical distance and environmental factors affect population genetic differentiation in the rice landraces of Yunnan province, China. Furthermore, the role of geographical or environmental isolation in genetic differentiation of rice landraces is still unclear.

We first collected 28 populations (594 individuals) of rice landraces that cover a wide geographical area and diverse growing conditions in Yunnan province, China, and analyzed ten gene sequences and 48 SSR (simple sequence repeat) markers. Then, a core subset of the collection of 594 landraces (108 accessions) was sampled for whole-genome sequencing. Our objectives were to examine the genetic structure and differentiation in rice landraces from Yunnan province, China, and to determine the relationships with their spatial and geographical distribution. We also discuss the role of geographical isolation and environmental heterogeneity in genetic differentiation among populations of rice landraces to provide theoretical support and a scientific basis for the protection and utilization of rice landraces.

## Results

### Genetic diversity

We sequenced ten unlinked gene segments from 594 accessions from 28 rice landrace populations in Yunnan (Table S1 and Figure S1). The length of the aligned sequences ranged from 420 to 627 bp for each gene locus, with a total length of 4,994 bp (Table S2 and Figure S2). The standard statistics of sequence polymorphisms for each locus are summarized in Table S3. The term θ_π_ indicates the nucleotide diversity, which was calculated for each locus. This term ranged from 0.0023 (*STS22*) to 0.0149 (*CatA*) in rice landraces, with an average of 0.0060. The haplotype diversity varied from 0.501 (*Os1977*) to 0.788 (*GS5*), with an average of 0.655. Four-gamete testing revealed that the Rm ranged from 0 to 7 in rice landraces, with an average of 3, indicating that there was low heterozygosity in the rice landraces due to high levels of inbreeding. Neutrality test values for most gene loci were not significant, except *Os1977* (*D* = 2.856) and *Ehd1* (*D* = 2.280), indicating that most were neutral gene loci. Based on SSR markers, a total of 629 alleles were detected in all accessions, with an average of 13.10 per locus. The average gene diversity and PIC were 0.755 and 0.725, respectively (Table S4).

**Fig. 1:**
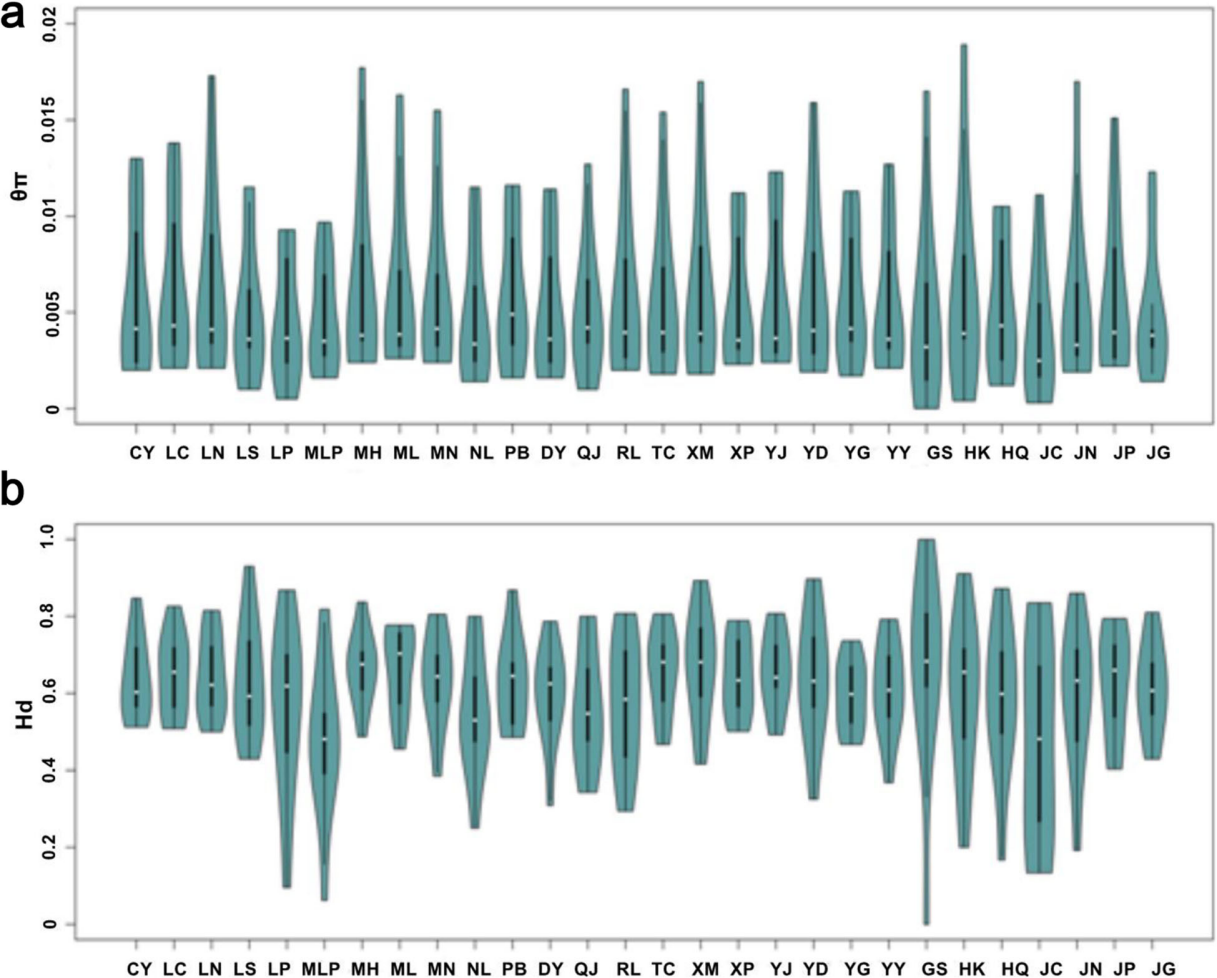
Violin plot of *θ*_π_ (**a**) and Hd (**b**) values for rice landraces from 28 populations

Standard sequence polymorphism statistics for each location are summarized in Table S5 and Fig. 1. For the gene loci, we observed genetic diversity, estimated by θ_π_, ranging from 0.0039 (Jianchuan, JC) to 0.0063 (Longchuan, LN), with an average of 0.0055. The haplotype diversity varied from 0.475 (Jianchuan, JC) to 0.671 (Ximeng, XM) (with an average of 0.610). Based on SSR markers, gene diversity, PIC, and heterozygosity ranged from 0.5082 to 0.7289, 0.4717 to 0.6928, and 0.0355 to 0.1264, respectively. Globally, the mean genetic diversity, PIC, and heterozygosity were 0.6729, 0.6318, and 0.0690, respectively. Correlation analysis indicated that genetic diversity was significantly negative with respect to latitude (Figure S3). In other words, the genetic diversity of rice landraces in Yunnan province, China showed a geographical decline from south to north.

### Population structure and genetic relationships

**Fig. 2:**
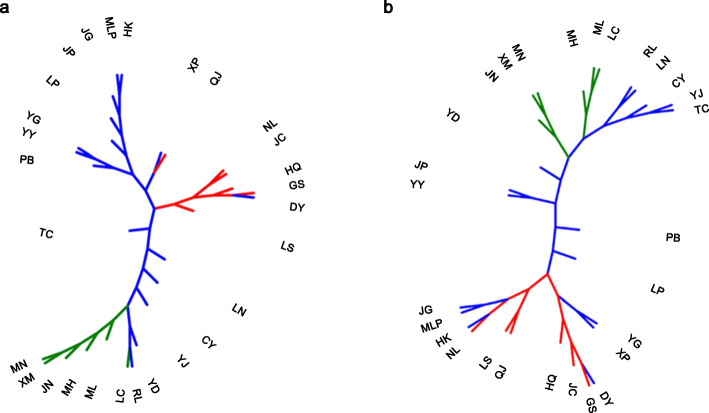
Unrooted neighbor-joining trees of the rice landraces from 28 locations based on Nei’s genetic distances in *japonica* (**a**) and *indica* (**b**) groups. Colours for rice landraces collected from different locations: red, from northern locations (N); green, from southern locations (S); blue, from middle locations (M)

The STRUCTURE analysis suggested K = 2 as the optimal number of clusters based on the calculation of ∆K (Figure S4). This suggests that the rice landraces can be grouped into two groups, referred to as P1 and P2. We found the 271 P1 accessions are *japonica* or *japonica*-like, and the 323 P2 accessions are *indica* or *indica*-like. This result indicated that rice landraces from Yunnan were clearly differentiated into *japonica* and *indica* groups (Figure S5a). We found both *japonica*- and *indica*-type landraces distributed in each location (Figure S5b). We further detected a significant positive correlation between the proportion of *japonica* rice landraces in various locations and latitudes (*r* = 0.367, *P* < 0.05), but a significant negative correlation between the proportion of *indica* rice landraces in various locations and latitudes (*r* = -0.367, *P* < 0.05) (Figure S6). In other words, the proportion of *japonica* landraces in each location in Yunnan increased from low to high latitudes. By contrast, the proportion of *indica* landraces in each location decreased from low to high latitudes. In each *japonica* or *indica* group, the neighbor-joining tree showed that the rice accessions could be clearly differentiated according to their geographic locations, including a cluster from northern locations, a cluster from southern locations, and other cluster from middle locations (Fig. 2). Moreover, we found more obvious geographic structure in the *japonica* group than in the *indica* group.

**Fig. 3:**
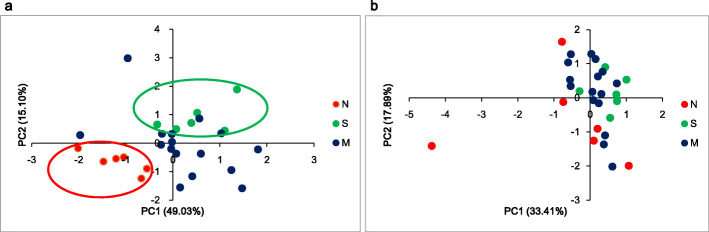
Principal components analysis (PCA) of the rice landraces from 28 locations based on phenotypic data in *japonica* (**a**) and *indica* (**b**) groups

In the *japonica* and *indica* groups, the first two components of the PCA accounted for 64.1 and 51.3 % of total variation, respectively, which were used to visualize the dispersion of the populations in a graph (Fig. 3). The result of PCA also showed geographic structure in the *japonica* group: we found that the rice accessions from northern locations were assigned to one cluster, the rice accessions from southern locations were assigned to one cluster, and rice accessions from locations in the middle were assigned to one cluster (Fig. 3a). In the *indica* group, we found less obvious geographic structure than in the *japonica* group, which was consistent with neighbor-joining tree (Fig. 3b).

### Isolation by distance and environment

**Fig. 4:**
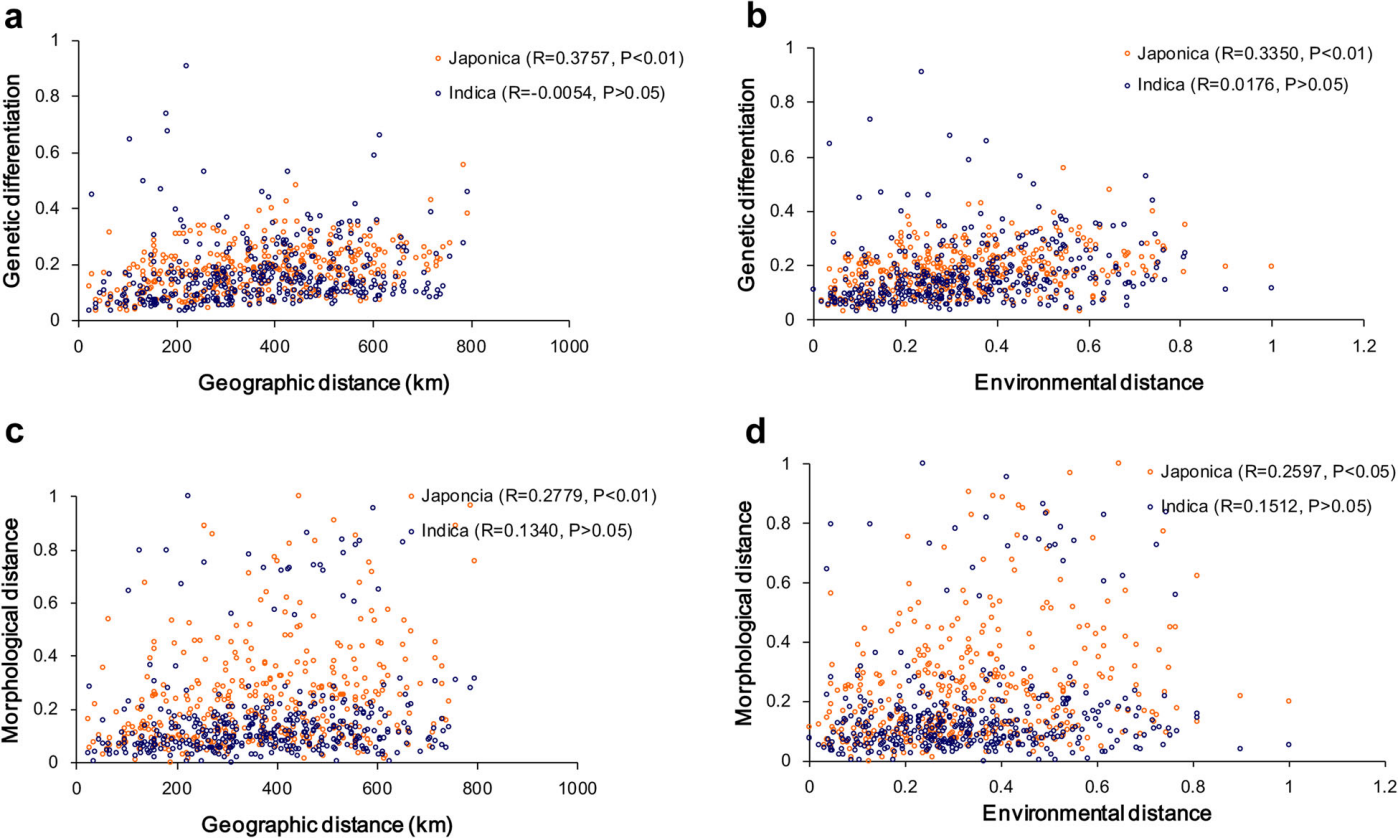
Patterns of isolation by distance (**a**, **c**) and by environment (**b**, **d**) across the rice landraces from 28 locations

In the *japonica* group, there was an overall relationship between pairwise genetic differentiation and geographic distance matrices (Mantel test, *R* = 0.3757, *P* < 0.01; Table 1; Fig. 4a), suggesting a pattern of IBD, and this relationship remained highly significant after controlling for environmental effects (partial Mantel test, *R* = 0.3579, *P* < 0.01; Table 2). Similarly, genetic differentiation was strongly related to environmental distance (*R* = 0.3350, *P* < 0.01; Table 1; Fig. 4b), suggesting a pattern of IBE; this relationship remained highly significant when we controlled for geography-level effects (*R* = 0.3142, *P* < 0.01; Table 2). For the individual gene loci, we found significant correlations between genetic differentiation and geographical distance regardless of whether we accounted for environmental effects at only the *GS5* locus (*R* = 0.3408, *P* < 0.01 and *R* = 0.3404, *P* < 0.01 respectively; Tables 1 and 2). Additionally, the positive genetic-environmental association was significant regardless of whether we accounted for geographical distance at only the *STS90* locus (*R* = 0.2114, *P* < 0.05 and *R* = 0.2223, *P* < 0.05 respectively; Tables 1 and 2). In the *indica* group, genetic differentiation was not related to geographic distance, regardless of whether environmental effects were accounted for (*R* = -0.0054, *P* > 0.05 and *R* = -0.0076, *P* > 0.05 respectively; Tables 1 and 2). Similarly, genetic differentiation was not related to environmental distance, regardless of whether geography-level factors were accounted for (*R* = 0.0176, *P* > 0.05 and *R* = 0.0184, *P* > 0.05 respectively; Tables 1 and 2).

**Table 1:** Mantel tests for the correlation between genetic/morphological differentiation and geographic/environmental distance of rice landraces in *japonica* and *indica* groups

Group	Matrix pair	Phenotype	All loci	SSR	*CatA*	*GBSSII*	*Os1977*	*STS22*	*STS90*	*S5*	*Pid3*	*Ehd1*	*GS3*	*GS5*
*Japonica*	G ×Dist	0.2779**	0.3757**	0.4075**	0.0077	0.0205	0.0147	0.0493	-0.0703	0.1081	0.1252	-0.0558	0.1309	0.3408**
	G ×Env	0.2597*	0.3350**	0.3280**	-0.0321	0.0937	0.1461	0.1355	0.2114*	0.0659	-0.0609	0.0672	-0.0362	0.0248
*Indica*	G ×Dist	0.1340	-0.0054	0.1557	0.0086	0.2258**	0.1078	0.1521**	-0.0105	0.1148	-0.0277	0.1394*	0.0518	-0.0412
	G ×Env	0.1512	0.0176	0.1562	0.1713	0.2676**	0.0986	0.0236	-0.0338	-0.1108	0.0484	-0.0234	-0.0450	0.009

*G* genetic distance; *Dist* geographic distance; *Env* environmental distance. *P < 0.05; **P < 0.01

**Table 2:** Partial Mantel tests of association between genetic/morphological differentiation and geographic/environmental distance of rice landraces in *japonica* and *indica* groups

Group	Matrix pair	Controlled	Phenotype	All loci	SSR	*CatA*	*GBSSII*	*Os1977*	*STS22*	*STS90*	*S5*	*Pid3*	*Ehd1*	*GS3*	*GS5*
*Japonica*	G ×Dist	Env	0.2568**	0.3579**	0.3919**	0.0118	0.0091	-0.0032	0.0332	-0.0991	0.101	0.1339	-0.0647	0.1364	0.3404**
	G ×Env	Dist	0.2367*	0.3142**	0.3069**	-0.0333	0.0919	0.1454	0.1306	0.2223*	0.0534	-0.0774	0.0747	-0.0531	-0.0181
*Indica*	G ×Dist	Env	0.1178	-0.0076	0.1393	-0.0079	0.2061**	0.0984	0.1505**	-0.0074	0.1267	-0.0324	0.1423*	0.0543	-0.0417
	G ×Env	Dist	0.1371	0.0184	0.1399	0.1712	0.2515*	0.0881	0.0093	-0.0329	-0.1231	0.0513	-0.0372	-0.0479	0.0112

*G* genetic distance; *Dist* geographic distance; *Env* environmental distance. *P < 0.05; **P < 0.01

Like genetic differentiation, population-level pairwise morphological distance was significantly related to geographic/environmental distance (*R* = 0.2779, *P* < 0.01; *R* = 0.2597, *P* < 0.05; Fig. 4c and d), and this relationship remained significant after controlling for environmental/geographic effects (*R* = 0.2568, *P* < 0.01; *R* = 0.2367, *P* < 0.05; Table 2) in the *japonica* group. However, in the *indica* group, morphological distance was not related to geographic distance between populations (*R* = 0.1340, *P* > 0.05; Table 1), nor to environmental distance (*R* = 0.1512, *P* > 0.05; Table 2).

To further demonstrate gene flow among different populations, we estimated frequency changes of dominant haplotypes at the important gene *Ehd1* (Early heading date 1) in different locations (Figure S7). The total number of haplotypes for *Ehd1* was 15 and 18 in the *japonica* and *indica* group, respectively. Among these haplotypes, H_3 was widely distributed in Yunnan in the *japonica* group, whereas H_1 was widely distributed in the *indica* group, which indicates that these two haplotypes were favored in *japonica* or *indica* group. In addition, we found there were some distinct differences in haplotype frequency among rice landraces from different locations. For example, the frequency of H_1 varied from 7.69 % (Luoping, LP) to 80.00 % (Qiaojia, QJ) (with an average of 34.57 %) in the *japonica* group, and the frequency of H_3 varied from 5.88 % (Yuanyang, YY) to 66.67 % (Luoping, LP) (with an average of 21.45 %) in the *indica* group. Notably, there were some environment-specific haplotype which could be found only in one location, such as H_2, H_11 and H_14 in the *japonica* group, and H_2, H_4 and H_6 in the *indica* group.

### Differential selection in the different geographic locations

**Fig. 5:**
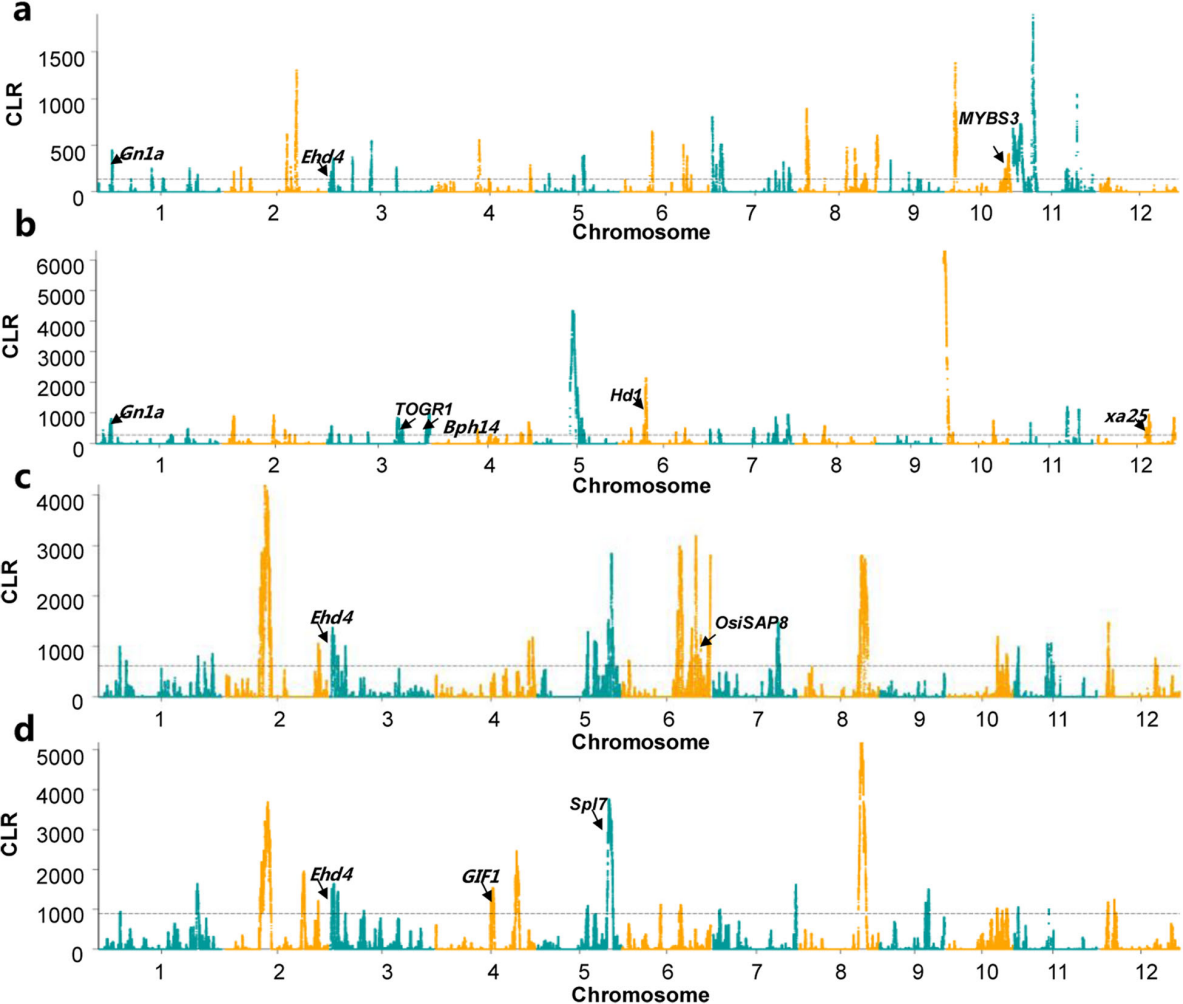
Genomic regions with strong selective-sweep signals in subgroup Jap-N (**a**), Jap-S (**b**), Ind-N (**c**) and Ind-S (**d**), respectively

We used a composite likelihood ratio method (CLR) (Nielsen et al. 2005) to identify genomic regions differentially selected in the subgroups from different geographic locations. (Fig. 5). Regions with the strongest 5th percentile of CLR selection signals were considered (Nielsen et al. 2005). In the *japonica* group, 179 regions and 170 regions were identified as the most affected by selection in subgroup Jap-N (rice landraces from northern locations) and Jap-S (rice landraces from southern locations), respectively (Table S6-7). The selected regions of Jap-N had a mean size of 105.9 kb, covering 5.1 % of the rice genome, whereas those selected regions of Jap-S covered 5.0 % of the rice genome with a mean size of 110.95 kb. The selected regions of Jap-N and Jap-S contained 2,165 and 1,785 protein-coding genes, respectively (Table S6-7). In the *indica* group, we identified 140 potential selected regions with an average size of 134.7 kb in Ind-N (rice landraces from northern locations), comprising approximately 5.0 % of the rice genome, and 77 potential selected regions with an average size of 234.5 kb in Ind-S (rice landraces from southern locations), comprising approximately 4.8 % of the rice genome (Table S8-9). The selected regions of Ind-N and Ind-S contained 1,964 and 1,875 protein-coding genes, respectively (Table S8-9). Thirty-seven selected regions of Jap-N overlapped with 35 selected regions of Jap-S, containing 309 genes, and 52 selected regions of Ind-N overlapped with 37 selected regions of Ind-S, containing 984 genes. These results suggested that although different target genes were selected in different subgroups, some of the targets were common to the different subgroups.

As expected, we found a number of candidate genes included in the potential selected regions were important agronomic genes/quantitative trait loci (QTLs) released by Q-TARO (Yamamoto et al. 2012) (Table S10-11). We found the gene *MYBS3* included in the selected regions of Jap-N on chromosome 10 (21.796–22.338 Mb, CLR = 332.87) (Fig. 5a), which were involved in cold stress response and cold tolerance (Su et al. 2010). Meanwhile, the region on chromosome 6 containing *OsiSAP8* showed a selective-sweep signal (24.469–24.593 Mb, CLR = 1148.06) in Ind-N (Fig. 5c). This gene was also related to cold tolerance (Kanneganti and Gupta 2008). Whereas, the selection signals were detected in regions of Jap-S and Ind-S, which includes the *TOGR1* and *Spl7* genes (Fig. 5b and d). These genes are involved in response to high temperature (Wang et al. 2016; Yamanouchi et al. 2002). Additionally, many important genes such as *Hd1* (Yano et al. 2000), *Ehd4* (Gao et al. 2013), *Gn1a* (Ashikari et al. 2005), *GIF1* (Wang et al. 2008), *xa25* (Chen et al. 2002) and *Bph14* (Du et al. 2009) were found in selected regions (Table S10). In total, we found 208 cloned agronomic genes in selected regions of Jap-N, Jap-S, Ind-N and Ind-S. Notably, these selective-sweep regions were extremely enriched in grain yield and plant type gene categories, followed by the abiotic stress resistance category (Figure S8). Furthermore, nearly all (96.6 %) of our selected regions were overlapped with previously reported agronomic QTLs (Table S11).

### Genomic signatures of local adaptation

To screen genomes for signatures of local adaptation, we tested for associations between genetic variation and environmental variables using Latent Factor Mixed Models (LFMM) (Frichot et al. 2013). A total of 1346 SNPs that obtained |z|-scores greater than 6 (*P* < 10^− 10^) (Table S12) were associated with environmental variables. Among these SNPs, we found the SNP (|z| = 7.02) located at 2.22 Mb on chromosome 3 was in the gene region of *sd37* involving in plant height in rice (Zhang et al. 2014), which indicated this candidate gene might be related to adaptation to the local environment (Table S13). We also found a significant SNP (|z| = 6.90) on chromosome 9 in the gene *OsMYBc* (Table S13), which is involved in salt tolerance (Wang et al. 2015), reflecting its adaption to the local environment. Among these SNPs significantly correlated with environmental variables, several notable examples include candidate genes involved in heading date (*OsRR1* and *OsRRM*) (Cho et al.^2016^; Chen et al. 2007), grain shape (*GL7* and *BG2*) (Yang et al. 2013; Wang et al. 2015), plant height (*CYP734A4* and *OsIAGLU*) (Choi et al. 2012; Qian et al. 2017), disease resistance (*OsCERK1* and *JAmyb*) (Cao et al. 2015; Huang et al. 2020), and heat tolerance (*HSP70*) (Breusegem et al. 1994), etc. (Table S13). All of these significant associations between loci/genes and environmental variables imply adaptation to the local environment. In total, in terms of the loci with high levels of association with environmental variables, we found 190 loci were also in the selected regions, which indicates that loci involving local adaptation have been particularly targeted in selection (Figure S9 and Table S12).

## Discussion

Owing to the wide geographical variation, diverse growing conditions, and cultural and ethnic diversity, Yunnan is acknowledged as one of the largest genetic diversity centers for rice in China and even worldwide (Zeng et al. 2001; Zhang et al. 2006; Zeng et al. 2007). In the present study, we analyzed the genetic diversity in rice landraces sampled from 28 locations that cover a broad geographical distribution including the diverse growing conditions in Yunnan province, China. We found the average haplotype diversity, gene diversity, and PIC were 0.655, 0.755, and 0.725, respectively (Table S3-4), which indicated there was a high level of genetic diversity in landraces from Yunnan and these landraces were extremely important for enriching the rice gene pool. We calculated the genetic diversity in each location measured by θ_π_ and PIC, which ranged from 0.0039 to 0.0063 and 0.4717 to 0.6928, respectively (Table S5), indicating that the patterns of genetic diversity are quite different geographically. Further analysis found the genetic diversity of rice landraces in Yunnan shows a geographic decline from south to north (Fig. S3). This result is consistent with the results of previous studies (Zhang et al. 2006; Zeng et al. 2007), which reported that the southwest region of Yunnan, encompassing the Simao, Lincang, and Xishuangbanna districts, is the center of genetic diversity for Yunnan rice landraces. In Yunnan, the common wild rice (*O. ruffipogon* Griff.), a direct ancestor of cultivated rice, is only found in Yuanjiang, Yuxi (the south-central region of Yunnan) and Jinghong, Xishuangbanna (the southwest region of Yunnan). Gene flow between landraces and their wild relatives greatly enhances the genetic variation of rice landraces in the genetic diversity center.

One important result of our study is the finding that genetic and phenotypic differentiation among rice landraces in the *japonica* group showed obvious geographic structure. However, we found less obvious internal geographic structure in the *indica* group. This result was consistent with a previous study: Xiong et al. found that the geographic distribution of *indica* and *japonica* rice landraces showed different patterns in Yunnan, China. The *indica* rice landraces with broad adaptability were widely distributed in the area between 2°S to 40°N, while the *japonica* rice landraces were obviously concentrated around 15°N (Xiong et al. 2011). Notably, a significant IBD pattern was revealed within the *japonica* group. The IBD pattern was further confirmed when the influence of environmental factors was considered (Table 2). This suggested that geographic isolation is one of the main factors influencing gene flow among the populations of the *japonica* rice landraces, implying that gene flow is more likely to occur between nearby locations. The pattern of IBD was highly associated with limited gene flow among populations due to affecting the spread of pollen and seeds. For inbred cultivated rice, where little to no pollen flow occurs, gene flow must occur by seed movement, and specifically by seed exchange among farmers (Pusadee et al. 2009). Farmers from the same or different locations establish networks and exchange their own seeds with others engaged in farming and cultural activities. In the ethnic minority regions of Yunnan province, seed exchange among indigenous farmers is also frequent. Seed exchange typically occurs between relatives, neighbors, friends, or through marriage patterns, and directly influences farmers’ seed exchange preferences whether within or between locations. Exchange among locations most often occurs between neighboring locations. Similar results were found in a previous study, where seed exchange between close communities played a role in reducing population structure in the rice landrace Bue Chomee in Thailand (Pusadee et al. 2009). Generally, differentiation occurs between locations, reflecting more limited seed exchange. For example, the local people in southern Yunnan are minority nationalities and live on rice. Daizu people dwell in the flatlands and enjoy eating glutinous rice, while Lahuzu people dwell in medium or highly mountainous regions and preferred red rice varieties. Therefore, seed exchange might be restricted between these locations under these circumstances, i.e., geographical isolation, traffic restriction, language, and custom barriers (Gao 2003).

Notably, our results revealed that gene flow among rice landrace populations also followed patterns of IBE for the *japonica* group, whereby gene flow rates are higher among similar environments. It indicated that environmental heterogeneity was essential in the genetic differentiation of *japonica* rice landraces in Yunnan as a result of limited dispersal. Additionally, the geographic distribution of haplotypes revealed the frequency changes of haplotypes at the important gene *Ehd1* (Early heading date 1) in different geographic locations and further demonstrated that both adaptation to local environment and environment-dependent gene flow played key roles in the genetic differentiation of rice landraces in Yunnan, China. Different environmental conditions (e.g., altitude, temperature, rainfall) can affect the survival and reproduction of plants and populations; thus, divergence might be due to local adaptation (Ohsawa and Ide 2008; Frei 2014). Local adaption may be enhanced with IBE. Under IBE, adaptation may be faster under stable environment conditions if populations from similar environments are exchanging beneficial alleles; however, with rapid environmental change, IBE could result in maladaptation if alleles from dissimilar environments are required for rapid adaptation (Sexton et al. 2014).

We next considered the potential role of natural and farmer selection in population genetic differentiation in rice landraces. We performed a genome-wide scan for signatures of selective sweeps to identify genomic regions differentially selected in the subgroups from different geographic locations. In the *japonica* group, 179 regions and 170 regions were identified as the most affected by selection in subgroup Jap-N and Jap-S, respectively. Similarly, we identified 140 and 77 potential selected regions in Ind-N and Ind-S, respectively. As expected, we found a number of candidate genes included in the potential selected regions and they were enriched in the grain yield category, followed by the plant-type and abiotic stress resistance categories, while enrichment was quite low in the eating quality category (Fig. S7). The results indicated selection for adaptation to each local environment results in rice landraces with high levels of local adaptation to abiotic and biotic stresses as well as for agricultural traits. For example, the rice landraces in Jap-N and Ind-N were from relatively high latitudes and we found that the genes involved in cold stress response and cold tolerance were included in the selected regions in these two subgroups. On the contrary, the rice landraces in Jap-S and Ind-S were from relatively low latitudes and we found that the genes involved in heat tolerance were included in the selected regions in these two subgroups. We also observed a number of significant and interesting associations between loci and environmental factors, which implies adaptation to local environment. Among these loci, we found 190 loci were also in the selected regions, which indicates that loci involving local adaptation have been particularly targeted in selection. Furthermore, different ethnic groups maintain and grow their own rice landraces for highly specific uses (e.g. ethnic dietary customs, medicinal uses, festivals, and religious ceremonies) (Gao 2003). The traditional cultures of these indigenous farmers has also resulted in population differentiation for the rice landraces of Yunnan.

## Conclusions

Our findings highlight the influence of geographical isolation and environmental heterogeneity on the pattern of the gene flow, and demonstrate that both geographical isolation and environmental heterogeneity were essential in the genetic differentiation of rice landraces in Yunnan province, China. Our study provides the theoretical support and a scientific basis for the protection and utilization of rice landraces.

## Methods

### Sample collection and choice of loci

We performed a large scale analysis of 594 rice landrace accessions collected from 28 sites that cover a wide geographical distribution and diverse growing conditions (Table S1). The sampled populations represent most of the genetic diversity present in rice landraces in Yunnan, China (Figure S1). We carefully collected between seven and 39 rice landrace accessions from each location based on the population size. In each location, we selected samples which can represent most of the diversity within Yunnan rice landraces in order to reduce sampling error as much as possible. All 594 accessions were studied using ten unlinked target genes and 48 microsatellite loci, and a core subset of the collection of 594 landraces (108 accessions) (Table S1) was sampled for whole-genome sequencing. The sequence data were released in our previous study (Cui et al. 2020).

A set of 48 SSRs with high polymorphism that map to loci evenly distributed throughout the rice genome (Table S14) and ten unlinked nuclear genes were used in this study. Five of the genes – *CatA*, *GBSSII*, *Os1977*, *STS22*, and *STS90* – had been used to estimate nucleotide diversity in rice populations in previous studies (Caicedo et al. 2007; Cui et al. 2016). We also used five additional genes – *Ehd1*, *Pid3*, *GS3*, *GS5*, and *S5* – that are known to be associated with environmental adaptation and important agronomic traits in rice (Doi et al. 2004; Chen et al. 2008; Shang et al. 2009; Mao et al. 2010; Li et al. 2011). Schematic diagrams of the structures of all ten genes are shown in Figure S2. Detailed information about the genomic location and putative functions of the genes, as well as the primer sequences for amplifying them, can be found in Table S2.

### DNA extraction, SSR genotyping, and gene sequencing

Total genomic DNA was extracted from fresh seedling leaves using a modified CTAB procedure (Doyle and Dickson 1987). The 48 SSRs were amplified by polymerase chain reaction (PCR) with fluorescently labeled primers in 10-µL reactions containing 20 ng of genomic DNA, 1 µl 10× PCR reaction buffer, 10 mM mixed dNTPs, 2 µM primers, and 0.5 units of Taq polymerase. The PCR profile consisted of a pre-denaturation step of 94 °C for 5 min, followed by 36 cycles of denaturation at 94 °C for 30 s, annealing at 55–60 °C (depending on primer sequences) for 30 s, and extension at 72 °C for 40 s, with a final extension at 72 °C for 10 min. PCR products were size separated on a 3730XL DNA Sequencer equipped with GENESCAN software (ABI, Waltham, MA, USA). Fragment sizes were recorded using Gene Marker V1.6 (SoftGene, State College, PA, USA) and manually re-checked.

To detect genes, PCR was performed in a 25-µL volume consisting of 0.2 µM of each primer, 200 µM of each dNTP, 10 mM Tris–HCl (pH 8.3), 50 mM KCl, 1.5 mM MgCl_2_, 0.5 U of HiFi DNA polymerase (Transgen, Beijing, China), and 10–30 ng of genomic DNA. The PCR amplification profile was as follows: pre-denaturation at 94 °C for 5 min, followed by 36 cycles of denaturation at 94 °C for 30 s, annealing at 55–60 °C (depending on the primers) for 30 s, and extension at 72 °C for 1.5 min, with a final extension at 72 °C for 10 min. The PCR products were electrophoresed on 1.2 % agarose gels, and DNA fragments were excised from the gel and purified using a Tiangen Gel Extraction Kit (Tiangen, Beijing, China). DNA sequencing reactions were performed using an ABI 3730 Automated Sequencer. Initially, all samples were directly sequenced. However, if the haplotypes could not be readily inferred owing to heterozygosity, the PCR products were ligated into a pGEM®-T Easy Vector (Transgen) and at least four clones were then sequenced. For heterozygous individuals, one allele sequence was randomly selected. Because Taq amplification errors can occur, when a polymorphism was found in only one accession, this accession was re-sequenced after cloning the amplified DNA fragment to verify the polymorphism.

### Analysis of DNA sequences and SSR data

DNA sequences were aligned using ClustalX 1.83 (Thompson et al. 1997) and edited using BioEdit 7.0.9.0 (Hall 1999). Indels were not included in the analysis. For each locus, we determined the number of segregating sites (S), the number of haplotypes (h), the haplotype diversity (Hd), and two nucleotide diversity parameters, mean pairwise differences (*θ*_π_) (Nei 1987) and Watterson’s estimator, based on the number of segregating sites (*θ*_w_) (Watterson 1975), using DnaSP version 5.0 (Rozas 2009). Statistical analysis of the SSR data, including allele number, genotype number, heterozygosity, gene diversity, and polymorphism information content (PIC), was performed using methods implemented in PowerMarker version 3.25 (Liu and Muse 2005).

### Population genetic structure

To identify population structure, a Bayesian clustering analysis was conducted using STRUCTURE 2.2 (Pritchard et al. 2000; Falush et al. 2003) based on data for the 48 SSRs. Fifteen independent runs were performed for each *k* value (from 1 to 12), using a burn-in length of 100,000, a run length of 100,000, and admixture and correlated allele frequency models. The *k* value was determined based on LnP(D) in the STRUCTURE output and the ad-hoc statistic Δ*k* (Evanno et al. 2005). We constructed a neighbor-joining tree using a matrix of pairwise genetic distances of 28 populations, calculated by PowerMarker version 3.25 (Liu and Muse 2005), and with tree visualization in MEGA 7.0 (Kumar et al. 2016). Principal component analysis (PCA) was performed with NTSYSpc software version 2.11 (Rohlf 2000).

### Isolation by distance and environment

Pairwise *F*_ST_ was calculated using Arlequin 3.5 (Excoffier and Lischer 2010). Population-level pairwise genetic differentiation as *F*_ST_/(1-*F*_ST_) (Slatkin 1995) was measured. Isolation by distance (IBD) was evaluated by assessing the correlation matrix between pairwise geographical distance and genetic differentiation between locations was assessed using Mantel tests. A total of 100,000 random permutations were performed. The analyses above were performed using methods implemented in Arlequin 3.5 (Excoffier and Lischer 2010 ).

We reduced the environmental variables using principal component analysis (PCA) based on the 24 environmental variables (Table S15) in SAS version 8.02. With PCA, we kept the first two axes as environmental variables because they explained 82.26 % of the environmental variation, and they were used to calculate the environmental dissimilarity. Prior to analysis, all environmental variables were standardized. The correlations between genetic differentiation and geographic/environmental factors were determined by partial Mantel tests. The environmental distance between populations was the Euclidean distance calculated with the values of the PCA axes. Partial Mantel tests with 100,000 permutations were performed between genetic factors and one factor under the influence of the other, using the “ecodist” package in R (Goslee and Urban, 2007). Because we identified two genetic clusters corresponding to the *indica* and *japonica* subpopulations (see results), partial Mantel tests were also conducted separately in each subpopulation.

### Morphometric analyses

To visualize the level of morphological structure across locations, we conducted a PCA of fifteen morphological traits (Table S16) and plotted the first two principal components (PC1 and PC2). We tested whether morphological differences between locations were the product of isolation by distance (IBD) or by environment (IBE) using the same rationale as for the genetic data. We calculated Euclidean distances in morphology between all pairs of locations, using the mean values per population of the fifteen morphometric traits (i.e. we obtained a single matrix of Euclidean distances in fifteen-dimensional space) (Table S16). Morphometric traits were standardized in the same manner as for environmental variables. We tested whether morphometric distance was related to geographic distance and environmental distance using Mantel tests and partial Mantel tests.

#### Selection analyses

A total of 2,771,245 high-quality SNPs of the representative 108 accessions were used for selection analyses. The sequence data were released in our previous study (Cui et al. 2020).

To detect regions with significant signatures of selective sweep, we performed a genome scan using a composite likelihood ratio method CLR (Nielsen et al. 2005) to calculate the candidate selected regions. A 10-kb sliding window across the whole genome was used for scanning. The highest CLR values, accounting for 5 % of the genome, were considered as selected regions.

#### Environmental association analysis

Latent factor mixed models (LFMM) (Frichot et al. 2013) were used to identify genetic variants associated with 3 environmental variables representing the 3 first components of a PCA for 24 environmental factors. The K-value was set to 2 based on the eigenvalues of the PCA of the genetic data as the number of latent factors. Five replicates were verified for convergence. The median z-scores of five runs was used to re-adjust the p-values. For an expected value of the FDR (q = 5 %), a list of candidate loci was obtained by using the Benjamini-Hochberg procedure.

## Supplementary Information

**Additional file 1: Figure S1. **Geographic localities of rice landraces sampled in this study. The localities of rice landraces are indicated by solid circles. Detailed information of the materials is provided in Table S1. **Figure S2. **Schematic diagrams of ten nuclear loci and locations of the sequenced regions. Exons are shown as open boxes and exon numbers are labeled with capital roman numbers. Thin lines between open boxes indicate introns. Locations of primers for each fragment are shown above the diagrams. **Figure S3. **Correlation between the number of haplotypes and latitude (a), between θ_π_ and latitude (b), between the number of alleles and latitude (c), and between gene diversity and altitude (d). **Figure S4. **The ΔK statistic for each given ***k***. **Figure S5. **Model-based ancestries and their distribution in each location. (a) Model-based ancestry of each accession in P1 and P2; (b) distribution of model-based populations in each location. **Figure S6. **Correlation between the proportion of ***japonica*** rice and latitude (a) and between the proportion of ***indica*** rice and latitude (b). **Figure S7.** A map showing the sampled populations of rice landraces and the distribution of haplotypes. (a) and (b) show rice landraces in the ***japonica*** and ***indica*** group, respectively. Phylogenetic relationship of the haplotype based on the NJ analysis is indicated below the map. Pie charts show the proportions of the haplotypes within each county. Haplotypes are indicated by different colors. **Figure S8.** Functional category of cloned genes in selected regions. **Figure S9.** “a” to “d” depict the composite likelihood ration (CLR) value of subgroup “Jap-N” (purple), “Jap-S” (orange), “Ind-N” (dark blue), and “Ind-S” (blue), respectively, and “e” presents |z|-scores of the SNPs which were tested for associations between genetic variation and environmental gradients using latent factor mixed models (LFMM).

**Additional file 2: Table S1.** List of samples included in the study, including their origin and group.** Table S2.** Summary of sequenced genes and primer sequences used in this study. **Table S3.** Summary of nucleotide polymorphisms and neutrality tests. **Table S4.** The genetic diversity of rice landraces based on 48 SSRs. **Table S5.** The genetic diversity of rice landraces in each population. **Table S6.** Putative selective-sweep regions in subgroup jap-N. **Table S7.** Putative selective-sweep regions in subgroup jap-S. **Table S8.** Putative selective-sweep regions in subgroup ind-N. **Table S9.** Putative selective-sweep regions in subgroup ind-S. **Table S10.** Cloned genes located on the selection-sweep regions. **Table S11.** QTLs that overlapped with selection-sweep regions. **Table S12.** SNPs with absolute value greater than 6 for the first three components of 24 environmental factors. **Table S13.** SNPs with the top 25 highest |z|-scores among those candidate genes with known functions. **Table S14.** Summary of SSR markers and primer sequences. **Table S15.** The detailed information of 24 environmental factors in each population. **Table S16.** Summary of agronomic traits for rice landraces in each population. The value before the slash shows the data collection from Yunnan, and the value after the slash shows the data collection from Hainan.

## Data Availability

All data supporting the conclusions of this article are provided within the article (and its additional files).

## Declarations

### **Data availability statement**

All data in this study have been uploaded as supplementary materials.
