# Supplementary material for: Genetic differentiation and restricted gene flow in rice landraces from Yunnan, China: effects of isolation-by-distance and isolation-by-environment
Source: Rice (N Y). 2021 Jun 15;14:54. doi: 10.1186/s12284-021-00497-6 (PMC8206287; doi:10.1186/s12284-021-00497-6)
Supplement: Supplementary file 1 — Additional file 1: Figure S1. Geographic localities of rice landraces sampled in this study. The localities of rice landraces are indicated by solid circles. Detailed information of the materials is provided in Table S1. Figure S2. Schematic diagrams of ten nuclear loci and locations of the sequenced regions. Exons are shown as open boxes and exon numbers are labeled with capital roman numbers. Thin lines between open boxes indicate introns. Locations of primers for each fragment are shown above the diagrams. Figure S3. Correlation between the number of haplotypes and latitude (a), between θπ and latitude (b), between the number of alleles and latitude (c), and between gene diversity and altitude (d). Figure S4. The ΔK statistic for each given k. Figure S5. Model-based ancestries and their distribution in each location. (a) Model-based ancestry of each accession in P1 and P2; (b) distribution of model-based populations in each location. Figure S6. Correlation between the proportion of japonica rice and latitude (a) and between the proportion of indica rice and latitude (b). Figure S7. A map showing the sampled populations of rice landraces and the distribution of haplotypes. (a) and (b) show rice landraces in the japonica and indica group, respectively. Phylogenetic relationship of the haplotype based on the NJ analysis is indicated below the map. Pie charts show the proportions of the haplotypes within each county. Haplotypes are indicated by different colors. Figure S8. Functional category of cloned genes in selected regions. Figure S9. “a” to “d” depict the composite likelihood ration (CLR) value of subgroup “Jap-N” (purple), “Jap-S” (orange), “Ind-N” (dark blue), and “Ind-S” (blue), respectively, and “e” presents |z|-scores of the SNPs which were tested for associations between genetic variation and environmental gradients using latent factor mixed models (LFMM). [file 12284_2021_497_MOESM1_ESM.pdf]

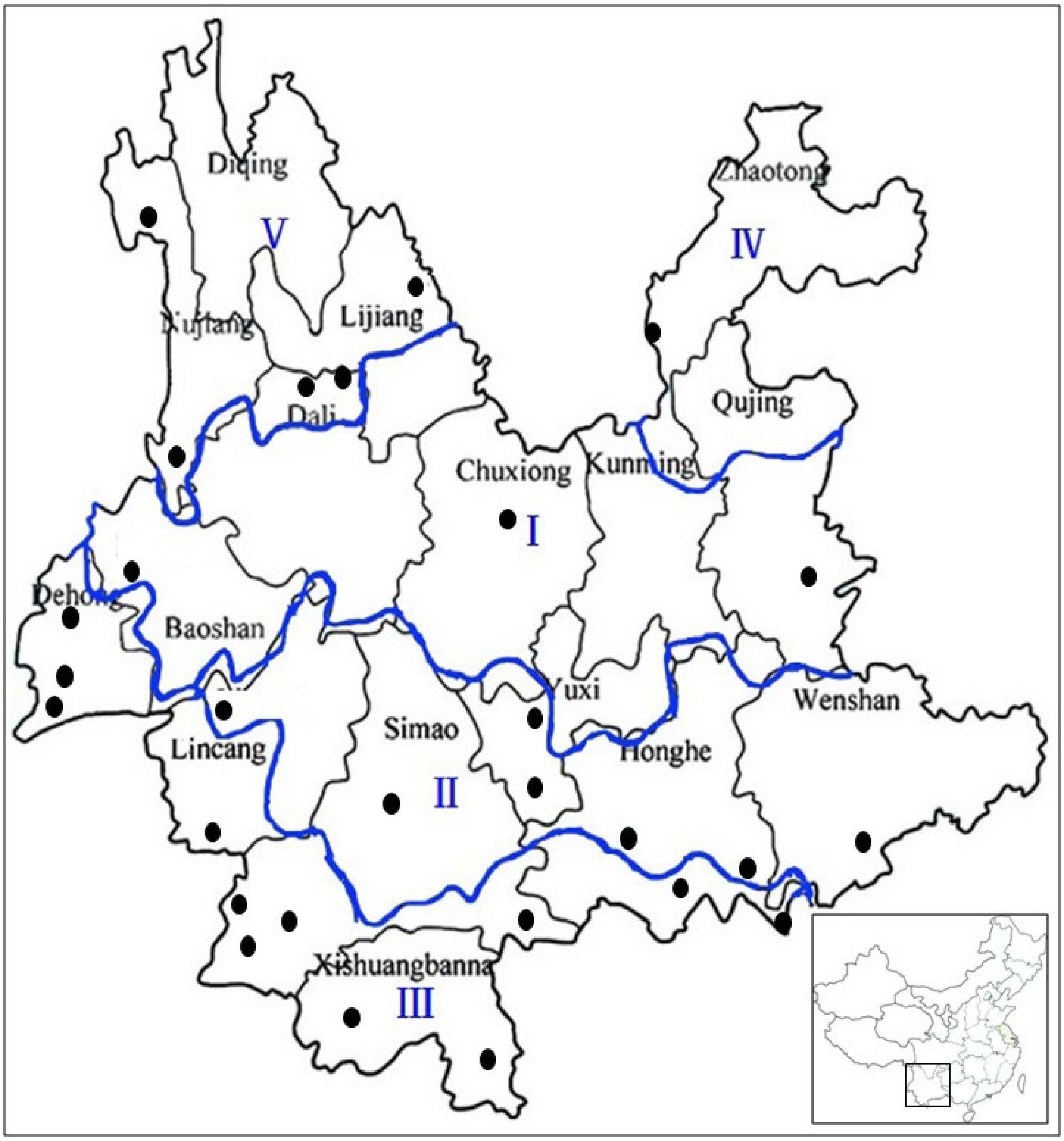

Fig S1. Geographic localities of rice landraces sampled in this study. The localities of rice landraces are indicated by solid circles. Detailed information of the materials is provided in Table S1.

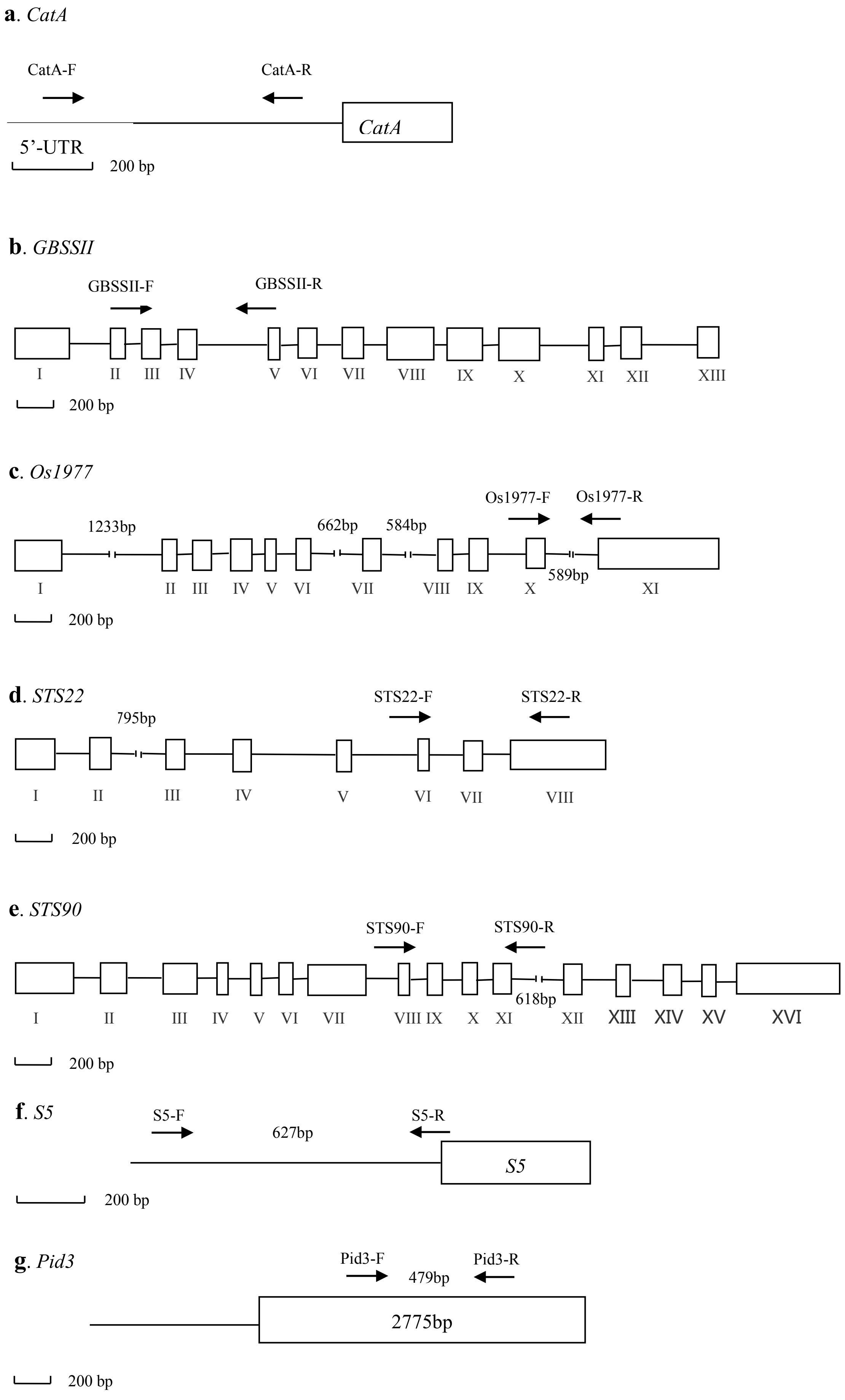

a. CatA
CatA-F
CatA-R
CatA
5'-UTR
200 bp
b. GBSSII
GBSSII-F
GBSSII-R
I
II
III
IV
V
VI
VII
VIII
IX
X
XI
XII
XIII
200 bp
c. Os1977
Os1977-F
Os1977-R
1233bp
662bp
584bp
589bp
I
II
III
IV
V
VI
VII
VIII
IX
X
XI
200 bp
d. STS22
STS22-F
STS22-R
795bp
I
II
III
IV
V
VI
VII
VIII
200 bp
e. STS90
STS90-F
STS90-R
618bp
I
II
III
IV
V
VI
VII
VIII IX
X
XI
XII
XIII
XIV
XV
XVI
200 bp
f. S5
S5-F
S5-R
627bp
S5
200 bp
g. Pid3
Pid3-F
Pid3-R
479bp
2775bp
200 bp

h. *Ehd1*

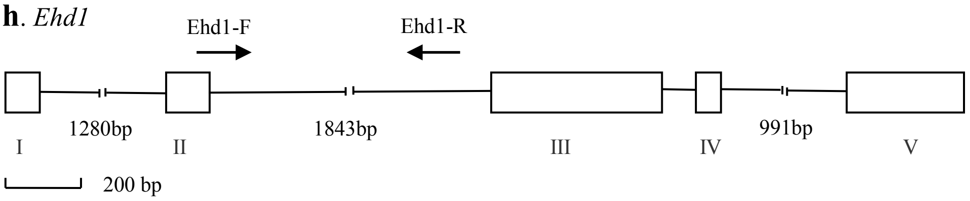

i. *GS3*

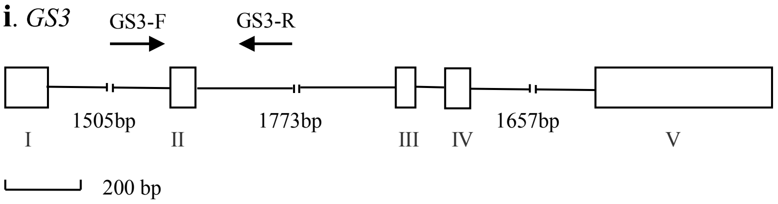

j. *GS5*

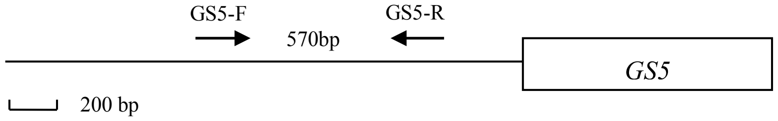

Fig S2. Schematic diagrams of ten nuclear loci and locations of the sequenced regions. Exons are shown as open boxes and exon numbers are labeled with capital roman numbers. Thin lines between open boxes indicate introns. Locations of primers for each fragment are shown above the diagrams.

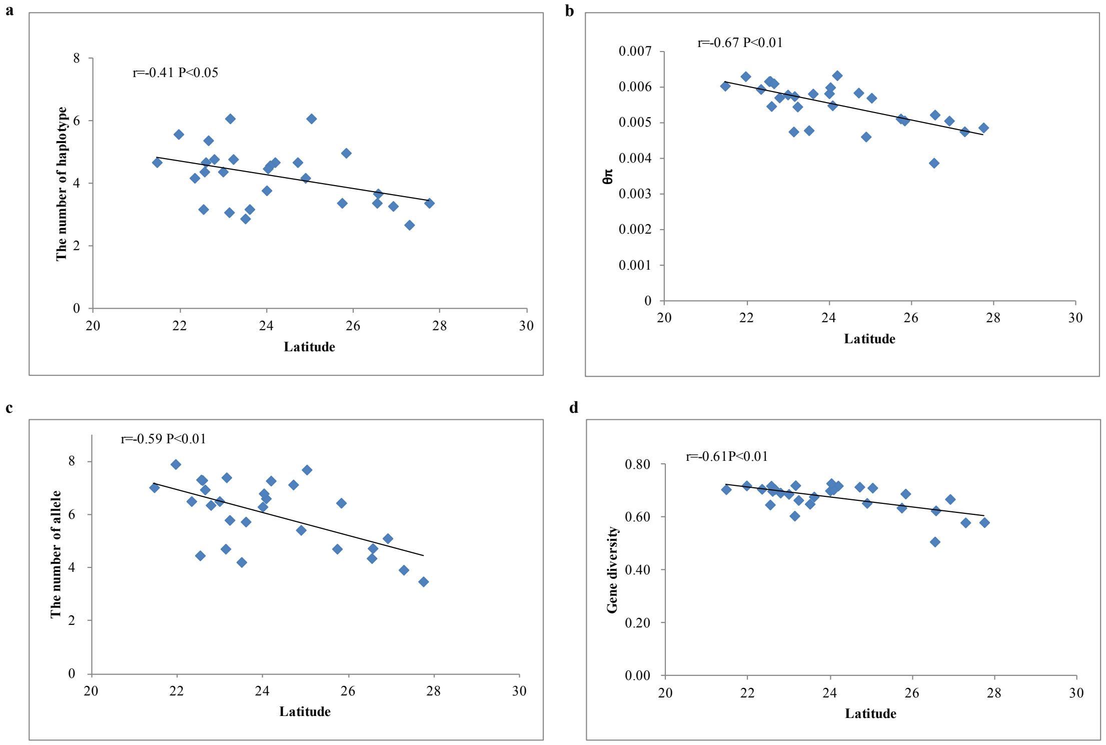

Fig S3. Correlation between the number of haplotypes and latitude (a), between θπ and latitude (b), between the number of alleles and latitude (c), and between gene diversity and latitude (d).

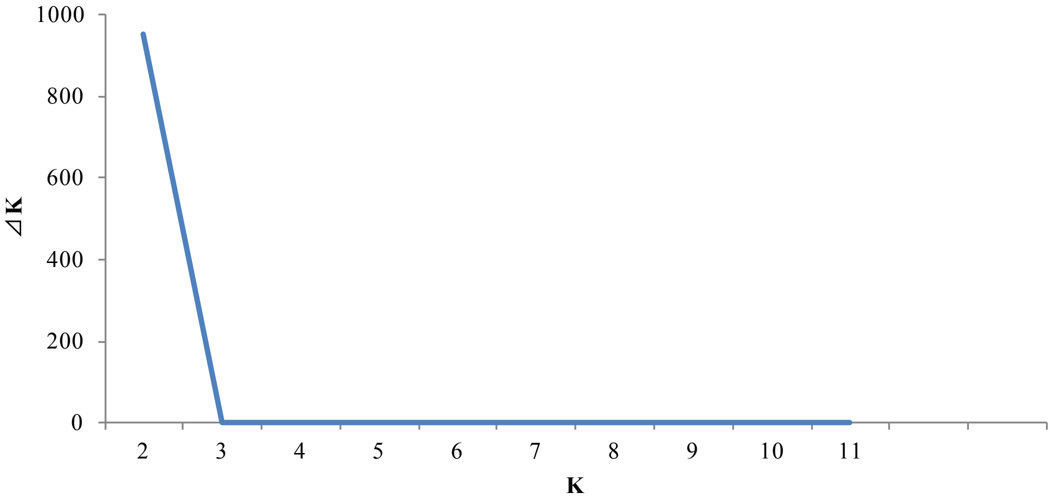

Fig S4. The ΔK statistic for each given *k*.

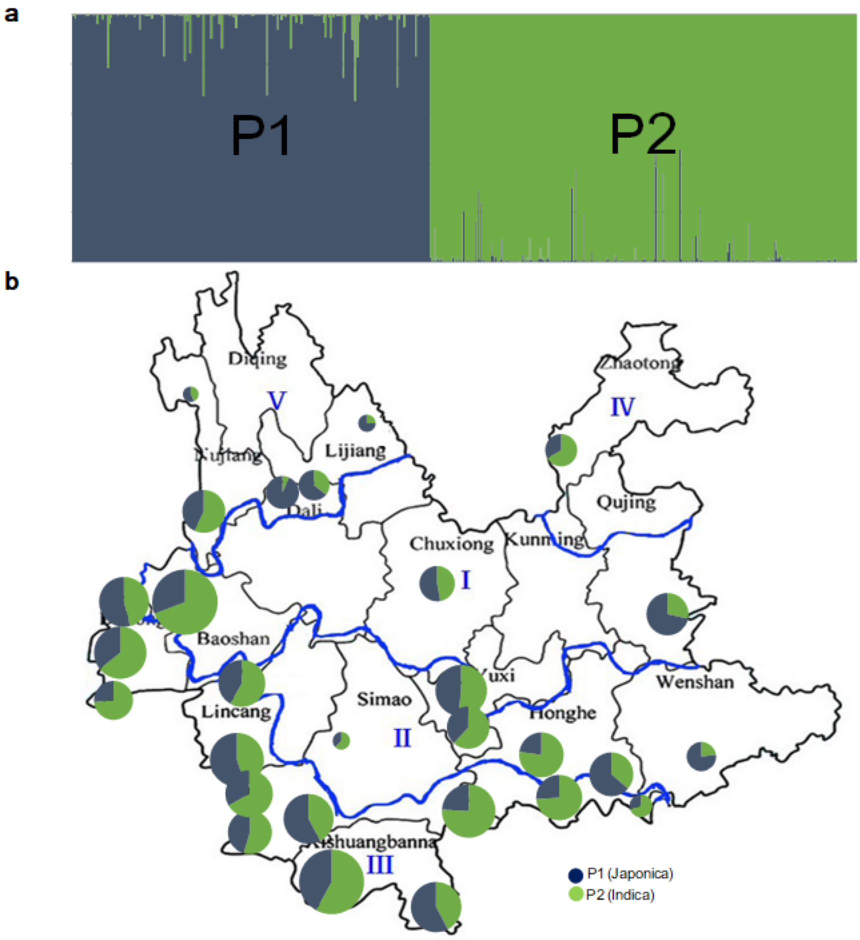

Fig S5. Model-based ancestries and their distribution in each location. (a) Model-based ancestry of each accession in P1 and P2; (b) distribution of model-based populations in each location

a

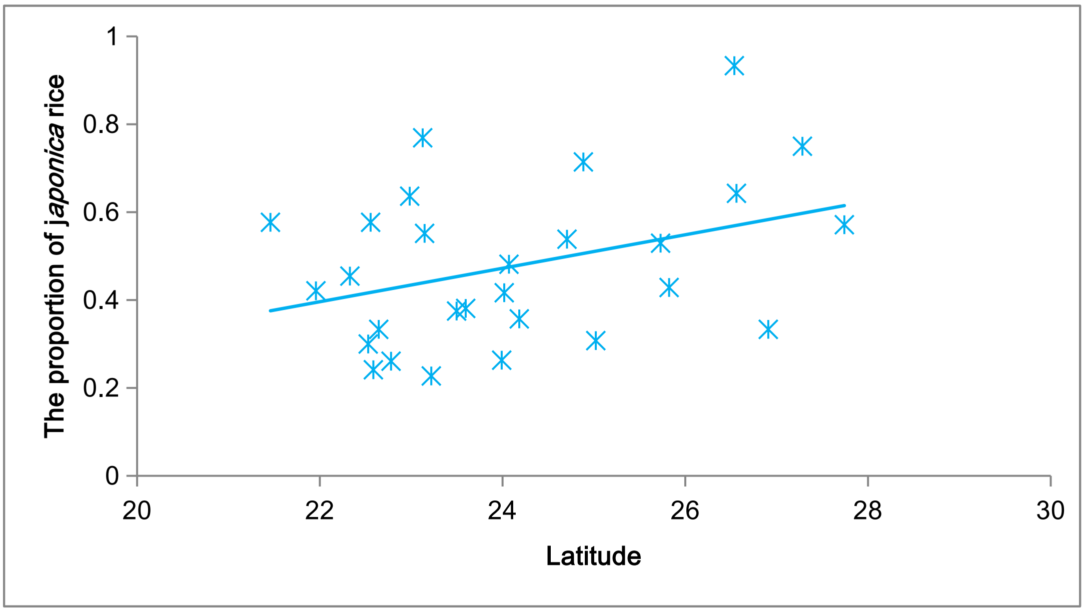

b

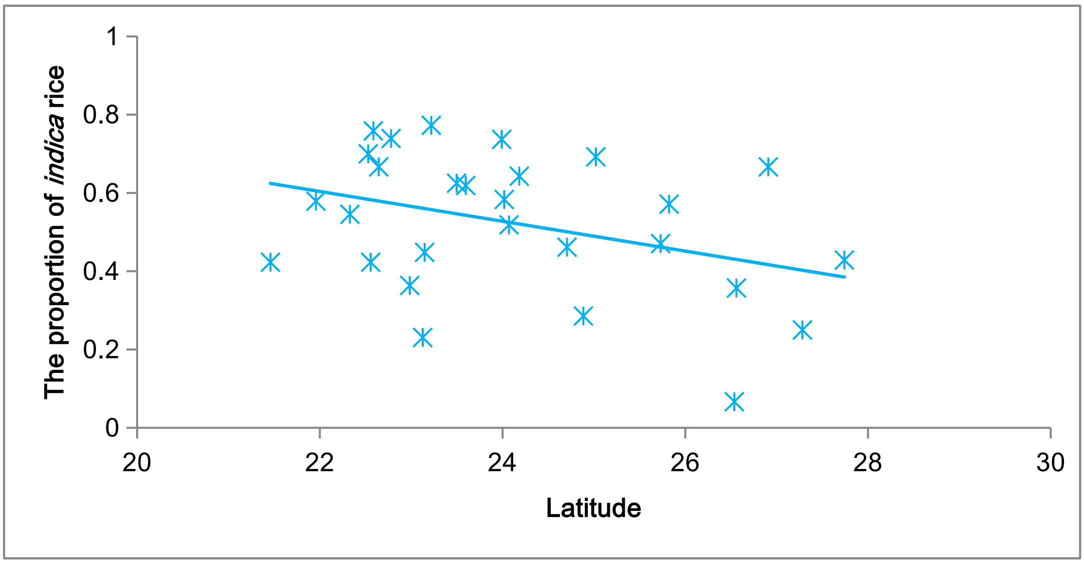

Fig S6. Correlation between the proportion of *japonica* rice and latitude (a) and between the proportion of *indica* rice and latitude (b).

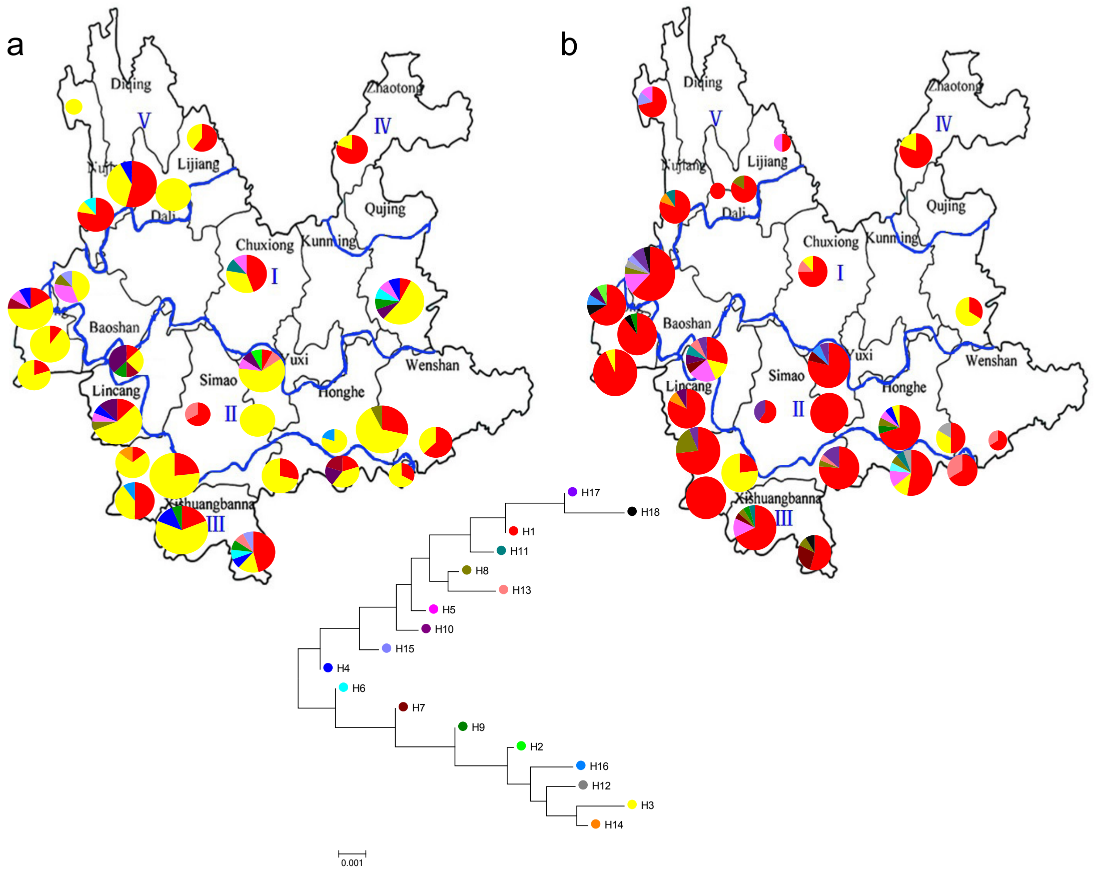

Fig S7. A map showing the sampled populations of rice landraces and the distribution of haplotypes. (a) and (b) show rice landraces in the *japonica* and *indica* group, respectively. Phylogenetic relationship of the haplotype based on the NJ analysis is indicated below the map. Pie charts show the proportions of the haplotypes within each county. Haplotypes are indicated by different colors.

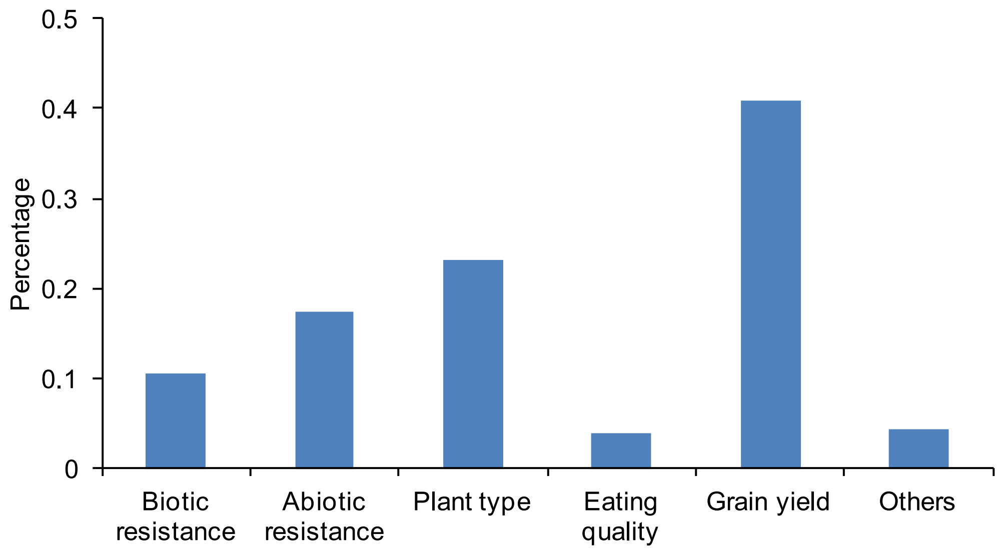

Fig S8. Functional category of cloned genes in selected regions.

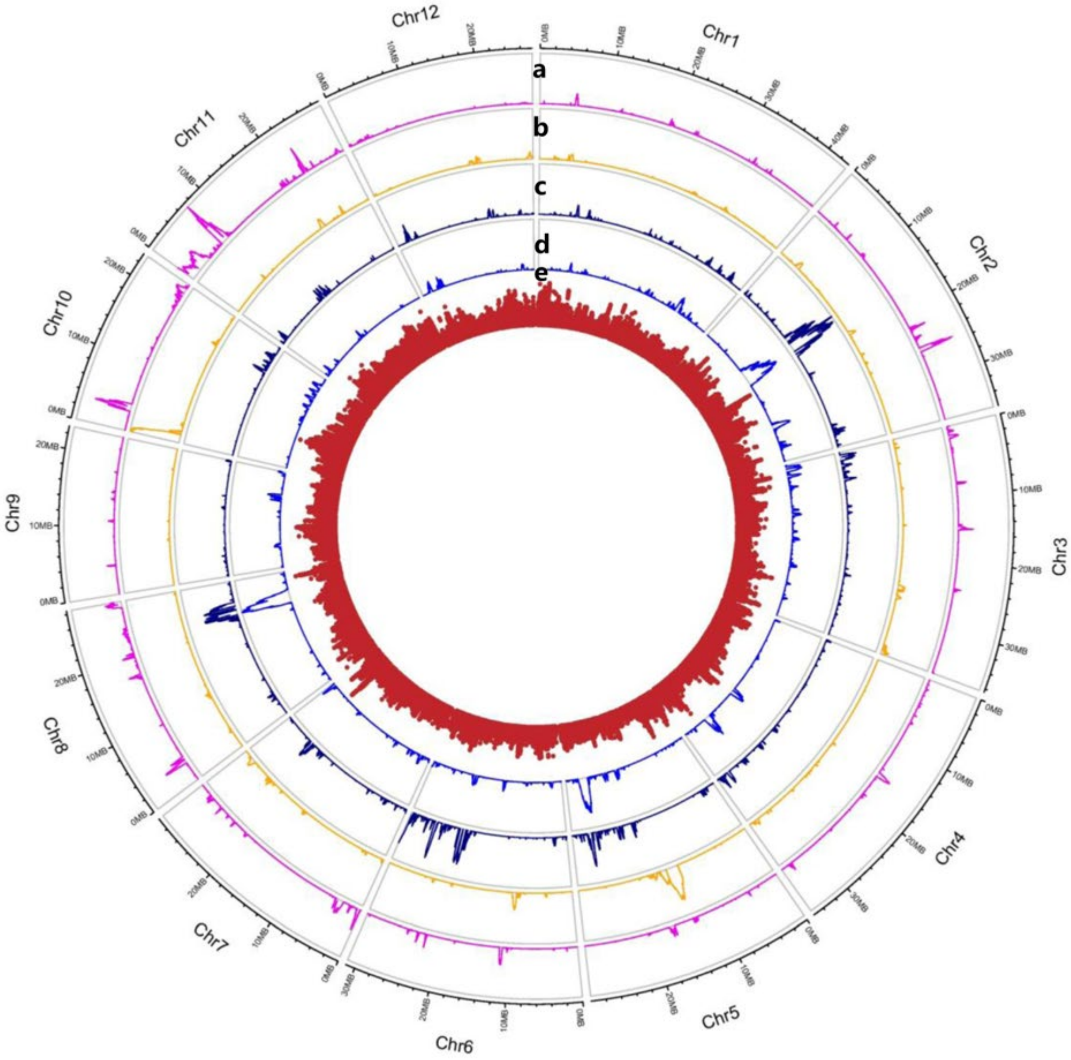

Fig S9. "a" to "d" depict the composite likelihood ration (CLR) value of subgroup "Jap-N" (purple), "Jap-S" (orange), "Ind-N" (dark blue), and "Ind-S" (blue), respectively, and "e" presents |z|-scores of the SNPs which were tested for associations between genetic variation and environmental gradients using latent factor mixed models (LFMM).
